# Chronic Hepatitis C: An Overview of Evidence on Epidemiology and Management from a Brazilian Perspective

**DOI:** 10.1155/2015/852968

**Published:** 2015-11-29

**Authors:** Rodolfo Castro, Hugo Perazzo, Beatriz Grinsztejn, Valdilea G. Veloso, Chris Hyde

**Affiliations:** ^1^Evandro Chagas National Institute of Infectious Diseases, Oswaldo Cruz Foundation, Laboratory of Clinical Research on STD/AIDS, Avenida Brasil 4365, 21040-900 Manguinhos, RJ, Brazil; ^2^Institute of Health Research, Peninsula Technology Assessment Group (PenTAG), Evidence Synthesis and Modelling for Health Improvement (ESMI), University of Exeter Medical School, University of Exeter, St Luke's Campus, South Cloisters, Exeter EX1 2LU, UK

## Abstract

Chronic hepatitis C remains one of the main causes of chronic liver disease worldwide and presents a variable natural history ranging from minimal changes to advanced fibrosis and cirrhosis and its complications, such as development of hepatocellular carcinoma. Approximately, 1.45 million people are estimated to be infected by HCV in Brazil representing a major public health issue. The aim of this paper was to review the epidemiology and management of chronic hepatitis C from a Brazilian perspective. The management of chronic hepatitis C has been challenged by the use of noninvasive methods to stage liver fibrosis as an alternative to liver biopsy and the high cost of new interferon-free antiviral treatments. Moreover, the need of cost-effectiveness analysis in hepatitis C and the recent changes in treatment protocols were discussed.

## 1. Introduction

Hepatitis C virus (HCV) was first described as non-A, non-B hepatitis in the 1990s in patients who presented with acute hepatitis after transfusion of blood products [1]. HCV is an enveloped RNA virus with 10 protein coding genes and a member of the family Flaviviridae, which targets hepatocytes leading to liver damage [2]. Parenteral transmission due to intravenous drug use, followed by transfusion of blood products before HCV screening, has been described as the most frequent route of infection. However, HCV can also be transmitted sexually or vertically [3]. Among patients exposed to HCV, a minority can spontaneously clear the virus [1] and around 85% of patients who still have detectable serum HCV RNA after 6 months should be considered as chronically infected [4].

Chronic hepatitis C (CHC) remains one of the main causes of chronic liver disease worldwide and presents a variable natural history ranging from minimal changes to extensive fibrosis and cirrhosis and its complications, such as development of hepatocellular carcinoma [5]. The correct determination of liver fibrosis stage has important implications for prognostic, therapeutic, and monitoring purposes [6]. Considerable progress in research and clinical practice has been made on hepatitis C infection since its discovery. Diagnosis and monitoring of infected patients were improved by molecular virological techniques and fibrosis staging using noninvasive methods. In addition, a promising new era in CHC treatment has been heralded by the recent approval of new drugs by licensing authorities around the world.

## 2. Diagnosis

Screening for HCV infection is performed by measuring anti-HCV antibody by serological enzyme immunoassay (EIA) that has been used as an indicator of past or present infection. The current third- and fourth-generation screening tests contain multiple recombinant antigens from HCV core and viral nonstructural proteins, such as NS3, NS4, and NS5, providing high accuracy for detection of exposure to HCV [7]. HCV antibodies can be detected by rapid diagnostic tests that use finger-stick whole-blood samples. These tests do not require complex instruments, yield the result in 20 minutes, and might be used in high burden HCV and low resources countries [8]. According to the recommendations from the US Center for Disease Control and Prevention (CDC) [9], all persons born between 1945 and 1965 or individuals presenting at least one of the following risk factors should be screened for HCV infection: patients who (i) have ever injected themselves with illegal drugs; (ii) received blood transfusion or organs transplant before the 1990s; (iii) have ever been in hemodialysis; (iv) have a persistently abnormal alanine aminotransferase (ALT) level; (v) were born to an HCV-positive mother; (vi) have human immunodeficiency virus (HIV) infection; and (vii) had a needle-stick injury or mucosal exposure to HCV-positive blood.

Patients with a positive anti-HCV should be tested for serum HCV RNA that quantifies the amount of RNA in serum and indicates ongoing HCV infection. Usually, HCV RNA is extracted from serum or plasma and amplified by polymerase chain reaction (PCR) [10]. Individuals with a positive HCV RNA should undergo disease staging to assess the extent of fibrosis by liver biopsy or noninvasive methods. In addition, the HCV genotype should be established in patients with CHC. Genotypes 1 to 6 are the most common in chronic viral infection, further divided into subtypes and strains. HCV genotyping is usually performed by direct sequencing of specific regions within the viral genome, followed by alignment to reference sequences and phylogenic analysis [11]. Genotype distribution varies according to geographic localization: HCV genotype 1 is the most frequent in the United States [12] and in Brazil [13, 14] and genotype 3 in India [15]. The definition of HCV genotype might guide the drug selection, dosage, and overall treatment duration.

## 3. Epidemiology of Chronic Hepatitis C

CHC is a disease that has global importance, since it was estimated to affect 2 to 3% of the world population, with 130–170 million people infected. The prevalence of HCV has a high variability between countries, with developed nations characterized by lower prevalence. A higher endemicity is present in countries of the African and the Eastern Mediterranean region [17, 18].

The Brazilian epidemiologic surveillance system uses a positive serum HCV RNA to define a confirmed case of hepatitis C infection. The compulsory notification of viral hepatitis started in 1996 in Brazil: 82,041 cases of HCV were notified, 60.1% in males and 39.9% in female patients from 1999 to 2011 [19]. Including the cases registered from 2012 to 2015, more than 150,000 confirmed HCV-positive patients were identified by the Brazilian surveillance system [20]. However, a very recently published report from Brazilian Ministry of Health estimated using mathematic modelling that around 1,450,000 people are living with HCV in Brazil [21]. These data justify all investments on HCV screening programs, implemented in order to reduce the number of people unaware of their HCV-positive status, and improve access to HCV-care. The quality of information about the infected patients will be improved with these testing strategies, reinforcement of the surveillance system, and research budgets offered for epidemiologic studies on HCV-prevalence in Brazil. The scarce data on HCV epidemiology, considering the real number of patients infected, is a problem in Brazil as in most of the Latin American countries [22].

The last extensive epidemiologic study was conducted from 2005 to 2009 using data from all major Brazilian cities. This research reported a prevalence of 1.38% (95% CI: 1.12%–1.64%) in 10–69-year-old subjects [23]. The predictors of HCV infection were age (OR = 1.01, 95% CI: 1.01–1.04), injected drug use (OR = 6.65, 95% CI: 2.47–17.91), sniffed drug use (OR = 2.59, 95% CI: 1.34–5.01), hospitalization (OR = 1.90, 95% CI: 1.03–3.51), and groups socially deprived by the lack of sewage disposal (OR = 2.53, 95% CI: 1.38–4.65) [23]. Genotype 1 was the most prevalent (79%) in a Brazilian study that aimed to evaluate the presence of cognitive dysfunction in CHC patients [13]. The higher prevalence of genotype 1 in Brazil was confirmed in a study that evaluated 283 patients: genotype 1 was present in 57.6%, genotype 3 in 39.6%, genotype 2 in 2.5%, and genotype 4 in 0.35% patients [14].

## 4. Prevalence of Liver Fibrosis and Cirrhosis and Its Complications in Brazil

The prevalence of liver fibrosis stages and cirrhosis was reported in small sample studies in Brazil. Using liver biopsy as the reference, mild to moderate fibrosis (F0F1F2) was observed in up to 70% of patients [13]. Fibrosis stages estimated by transient elastography were reported in 120 CHC patients: 54%, 30%, 9%, and 7% for METAVIR stages F0F1, F2, F, and F4, respectively [24]. These results were confirmed in a more recent study [25]. Up to 8% of Brazilian patients with cirrhosis developed hepatocellular carcinoma (HCC) in a 10-year follow-up period [26].

End-stage cirrhosis due to HCV is the main indication for liver transplantation worldwide [27]. Up to 75% of patients submitted to liver transplantation in a Brazilian tertiary hospital had CHC [28]. The number of liver transplants has been growing in Latin America, especially in Brazil: this country has achieved the third largest volume of liver transplantation in the world with more than 1,600 liver transplantations performed in 2012 [29].

## 5. Natural History

Most patients exposed to HCV cannot clear the virus spontaneously and develop chronic infection. The host's age, gender, and other comorbidities, such as body weight, hepatic steatosis, alcohol abuse, and coinfection with hepatitis B virus and/or HIV, might play a crucial role in the ability to spontaneously clear the virus [30]. The natural history of CHC is extremely variable ranging from minimal changes to extensive fibrosis, cirrhosis, and development of severe complications, such as end-stage liver disease, portal hypertension, and hepatocellular carcinoma [5]. The chronic disease is generally slowly progressive and cirrhosis develops within 20 years in about 10–20% of patients chronically infected [31]. The prevalence of cirrhosis after 20 years of HCV infection was estimated at 16% (95% CI 14–90%) in a systematic review that included 111 studies [32]. Similar results were reported in studies in France [33]. Several factors might influence the progression of liver disease, such as the age of acquisition, gender, coexisting viral disease particularly HBV or HIV coinfection, concomitant alcohol abuse, and the host immune response [34].

HCV infection might be associated with extrahepatic manifestations such as cryoglobulinaemia, vasculitis, lichen planus, porphyria cutanea tarda, lymphocytic sialoadenitis, and membranous glomerulonephritis [35]. In addition, CHC might be associated with non-Hodgkin's lymphoma [36]. The development of cirrhosis might be associated with major complications such as ascites, gastrointestinal bleeding, severe bacterial infection, and encephalopathy [37]. Cirrhotic patients with compensated disease have a risk of 1–4% per year of developing hepatocellular carcinoma and a risk of 4% per year of developing hepatic decompensation. Those with decompensated cirrhosis have a survival rate of 50% at 5 years [38].

## 6. Staging Liver Fibrosis

Accurate assessment of the fibrosis stage and the grade of necroinflammatory activity are essential for the management of patients with CHC. Historically, the severity of liver disease has been measured by liver biopsy using semiquantitative histological scoring systems, such as METAVIR [39] and Ishak score [40]. However, liver biopsy is a painful invasive method and might be associated with potential complications that range from local pain to intraperitoneal bleeding, associated in turn with transfusion of blood products, surgery, or death [41]. In Brazil, Bergesch D'Incao et al. reported 0.3% of severe complications in a study that evaluated 1955 liver biopsies in patients with chronic liver disease, most infected by viral hepatitis. In this study, the majority of severe complications were gallbladder perforation and all patients with complications were submitted to surgery after liver biopsy [42]. In addition, the usefulness of liver biopsy has been challenged by limited feasibility, adverse effects, sampling error, and interobserver variability [43–45].

Several noninvasive methods have been proposed to assess liver fibrosis in patients with chronic viral hepatitis as an alternative to liver biopsy: serological biomarkers and imaging techniques [46]. Few biomarkers, such as aspartate-to-platelet ratio index (APRI) or Fibrosis-4 score (FIB-4) are nonpatented tests that use simple and worldwide available parameters in their formula. Other biomarkers are patented and involve the combination of more complex biological parameters, such as hyaluronic acid, alpha-2-macroglobulin, or serum markers involved in the synthesis and breakdown of the extracellular matrix [47]. Transient elastography by FibroScan (EchoSens, Paris, France) is one of the most validated noninvasive methods based on imaging techniques [48].  Table 1 summarizes the several noninvasive methods currently available for estimation of liver fibrosis in chronic viral hepatitis.

Liver fibrosis can be estimated by APRI using simple and worldwide available serological parameters such as AST and platelet count. This biomarker can be calculated according to the following formula: AST level (/ULN)/Platelets count (10^9^/L) *∗* 100. Liver fibrosis, estimated by APRI, can be converted to the METAVIR scoring system [39] as proposed by Wai et al. [49] using dual cut-offs: >0.5 and >1.5 for fibrosis stage *F* ≥ 2 and >1.0 and >2.0 for cirrhosis (*F* = 4). In this first study, APRI yielded 0.91 of sensitivity and 0.47 of specificity for fibrosis stage *F* ≥ 2 and 0.89 of sensitivity and 0.75 of specificity for cirrhosis when the lower set of cut-offs >0.5 for *F* ≥ 2 and >1.0 for *F* = 4. In addition, this test yielded 0.41 of sensitivity and 0.95 of specificity for fibrosis stage *F* ≥ 2 and 0.57 of sensitivity and 0.93 of specificity considering the higher set of cut-off (>1.5 for *F* ≥ 2 and >2.0 for *F* = 4).

A potential criticism to this test could be the impact of necroinflammatory activity in the estimation of liver fibrosis due to the use of transaminases in its formula and the fact that upper limit of normal for AST is extremely variable from a laboratory to another depending on the control group used. This variability induces a spectrum effect, which could cause misleading interpretations of APRI performance for the staging of fibrosis [50].

The performance of APRI to stage liver fibrosis was validated in a systematic review yielding an area under the receiver-operating characteristic curve [AUROC (95% CI)] of 0.77 (0.58–0.95) for fibrosis stage *F* ≥ 2 and 0.84 (0.54–0.97) for cirrhosis (*F* = 4) [51]. APRI has also been reported to be predictive of mortality in CHC [52–54]. Few studies have been published regarding the diagnostic performance of APRI with data from Brazilian centers. Initially, Parise et al. reported the diagnostic performance of APRI in 206 patients with HCV infection: AUROC = 0.824 (0.772–0.903) for fibrosis stage *F* ≥ 2 [55]. In addition, da Silva Jr. et al. described AUROC of 0.92 (0.83–1.00) and 0.92 (0.85–1.00) for diagnosis of fibrosis stage *F* ≥ 2 and cirrhosis, respectively, using liver biopsy as the reference in a small sample (*n* = 41) of patients with CHC [56]. Following the same trend, Amorim et al. reported an AUROC of 0.793 ± 0.047 for APRI to estimate fibrosis stage *F* ≥ 2 in 119 HCV-infected patients [57] and, more recently, Silva Junior et al. reported AUROC of 0.82 for both diagnosis of fibrosis stage *F* ≥ 2 and cirrhosis [58].

Transient elastography (TE), assessed by FibroScan (EchoSens, Paris, France), is an imaging method that estimates liver fibrosis based on liver stiffness measurement (LSM) [59]. The examination is very well accepted by patients, is painless and fast (<10 minutes), and can be easily performed at bedside or in the outpatient clinic without potential complications. The performance of 100 exams can be used to define an experimented operator [60]. However, TE requires an expensive medical device operated by an experienced operator and this method has been challenged concerning its reproducibility and overestimation of fibrosis in special situations.

The performance of TE, expressed as AUROC, for diagnosis of fibrosis stage *F* ≥ 2 and cirrhosis (*F* = 4) varies from 0.79 to 0.83 and from 0.95 to 0.97 in HCV-infected patients. A cut-off value of 12.5 kPa yielded sensitivity, specificity, and AUROC values of 0.87, 0.91, and 0.95, respectively, for the diagnosis of cirrhosis [61], whereas a cut-off of 14.6 kPa yielded sensitivity, specificity, and AUROC values of 0.86, 0.96, and 0.97, respectively [62]. A meta-analysis validated the diagnostic performance of TE: for detection of cirrhosis, the summary sensitivity was 0.92 (95% CI: 0.78–0.97) and the summary specificity was 0.86 (95% CI: 0.82–0.90). TE has a prognostic value for 5-year prediction of overall mortality [53] and severe outcomes in CHC patients [63]. In Brazil, a single study has reported the diagnostic accuracy of TE: AUROC ranged from 0.87 to 0.94 for diagnosis of fibrosis stage *F* ≥ 2 and cirrhosis, respectively, in 120 patients with CHC, mostly with mild fibrosis (F0 or F1) [24]. Another two studies have evaluated the intra- and interobserver agreement of this method in a Brazilian population [25, 60].

## 7. Health-Related Quality of Life and Cost-Effectiveness of Noninvasive Diagnostic Tests for Surveillance of Liver Disease in Brazil

Studies have reported worse health-related quality of life (HRQOL) for people living with HCV in Brazil [64–67]. These results demonstrate that CHC has impact on the quality of life, which might be due to the HCV itself according to a study with blood donors unaware of the HCV-positive diagnosis [66]. CHC was associated with depression [68] and sexual dysfunction [69] and the diagnosis of HCV was frequently considered a traumatic experience [70]. Few studies used quality-adjusted life years (QALY) as an outcome measure to estimate effects of interventions for Brazilian patients [71, 72]. However, both studies have used international data for utilities estimation due to local information unavailability. Few studies have used cost-effectiveness analysis to evaluate interventions for CHC from a Brazilian perspective: treatment with Peg-interferon and Ribavirin and hypothetical vaccine should be cost-effective [71–74]. Similar results were reported for HCV/HIV coinfected patients [74].

Since CHC is a major public health issue with new costly drugs for treatment, leading to an economic impact on the healthcare systems [75], more health technology assessments for treating and preventing CHC in Brazil are urgently needed to tackle this public health issue. Moreover, cost-effectiveness analysis of noninvasive methods for staging liver fibrosis in CHC patients must be conducted to establish evidence-based criteria for treatment decisions.

## 8. Evidences on HCV-Treatment and the Promising New Brazilian Guidelines

The main objective of treatment of CHC remains eradication of HCV represented as sustained virological response (SVR) that has been associated with lower overall and liver-related mortality [76]. Several factors can influence the response rate, such as HCV genotype, stage of fibrosis, or results of previous HCV-treatment [77]. Treatment using Peg-interferon has been challenged by low response rates, especially in genotypes 1 and 4 and significant side effects [78].

Considering the effectiveness of retreatment, results from a Brazilian cohort of patients with HCV genotypes 2 and 3 showed 34.4% of SVR rate for nonresponders to previous treatment and 50% for relapsers with Peg-interferon combined with Ribavirin [79]. Other Brazilian cohort studies obtained 79.3% (46/58) of SVR for patients with HCV genotypes 2 or 3 treated with Peg-interferon plus Ribavirin and 49.1% (56/114) among those treated with biosimilar standard interferon plus Ribavirin [80].

In recent years, the standard of care has been replaced by new direct-acting antiviral drugs (DAAs) that act by inhibiting protease or nonstructural proteins of HCV. Currently, these drugs are in various stages of preclinical and clinical development. The new treatment regimens has been based on the combination of DAA that can be classified as protease inhibitor (first-generation: Boceprevir and Telaprevir; second-generation: Simeprevir, Paritaprevir), NS5A inhibitor (Ledipasvir, Daclatasvir, and Ombitasvir), or NS5B inhibitor (Sofosbuvir, Dasabuvir, and Beclabuvir) [81].

In 2011, the first-generation protease inhibitors were approved for clinical use. Patients with genotype 1 treated by a combination of Boceprevir or Telaprevir plus Peg-interferon and Ribavirin had a significantly higher SVR compared to those treated by the standard of care with Peg-interferon and Ribavirin [82, 83]. However, patients also suffered high rates of side effects [84] and several drug interactions [85] and viral resistance might occur and patients with cirrhosis need a longer duration treatment [86]. In 2014, new DAAs, such as Simeprevir (second-generation protease inhibitor), Daclatasvir (NS5A inhibitor), and Sofosbuvir (NS5B inhibitor), were licensed for treatment in combination with Peg-interferon and Ribavirin in genotype 1 patients. Peg-interferon plus Ribavirin in combination with Simeprevir [87] or Sofosbuvir [88] had a SVR in about 80% and 90% of patients, respectively. New interferon-free treatments will probably be the standard of care due to their high efficacy and low adverse events for untreated patients and previous interferon nonresponders. These regimens might comprise the following combinations: (i) protease or NS5A inhibitor plus a NS5B inhibitor with or without Ribavirin; (ii) a protease inhibitor plus a NS5A and a NS5B inhibitor with or without Ribavirin; or (iii) a protease inhibitor and a NS5A inhibitor with or without Ribavirin.

Several studies have evaluated the efficacy of interferon-free regimens in HCV genotype 1 patients. The combination of Sofosbuvir (NS5B inhibitor) with Ledipasvir (NS5A) in combined pill once daily without Ribavirin for 12 weeks is highly effective in untreated (SVR 97%) [89] and previously treatment-experienced patients (SVR 93%) [90]. In addition, the same regimen can achieve a 94% SVR in treatment-naive patients in 8-week treatment. The efficacy of Sofosbuvir/Ledipasvir with Ribavirin remains very high in patients with well-compensated cirrhosis (Child-Pugh-Turcotte class A) in a 12-week treatment (96% SVR) [90]. Other potentially effective oral combination regimens have been described. The combination of Paritaprevir (second generation protease inhibitor) boosted by Ritonavir with Ombitasvir (NS5A inhibitor) and Dasabuvir (NS5B inhibitor) in a combined pill twice daily with Ribavirin had a 96% SVR in treatment-naive genotype 1 patients and 96% of SVR in noncirrhotic nonresponders to Peg-interferon and Ribavirin [91]. Similar results were described in cirrhotic treatment-naive or treatment experimented patients treated for 12 (with 92% of SVR) or 24 weeks (96% SVR) [92]. In addition, the coformulation Sofosbuvir/Simeprevir had a 92–96% SVR depending on stage of fibrosis or previous treatment [93].

Similar results have been described in genotypes 2 and 3 patients. Sofosbuvir with Ribavirin is highly effective in 12-week treatment of genotype 2 (97%) but a longer treatment is needed in those with genotype 3. In addition, response rate is lower in those with cirrhosis [94, 95]. High rates of SVR (94–100%) have been reported in genotypes 2 and 3 patients treated with the combination Sofosbuvir/Daclatasvir [96]. Similar results were reported in a regimen combining Sofosbuvir and Ledipasvir for patients with genotype 3 [97]. Those combinations, Sofosbuvir/Daclatasvir and Sofosbuvir/Simeprevir or others such as Ritonavir boosted Paritaprevir/Ombitasvir, showed efficacy in patients with genotype 4 [98, 99].

The Brazilian Ministry of Health has recently approved guidelines for treatment of CHC with interferon-free regimens [16]. Sofosbuvir, Daclatasvir, and Simeprevir were included for interferon-free treatment. These drugs have been adopted by the Brazilian government and delivered free to patients with advanced fibrosis (stages *F* ≥ 3) by the public health system (SUS;* Sistema Único de Saúde*). Liver biopsy, serological biomarkers (APRI and FIB-4), or elastography methods have been accepted to stage liver fibrosis. Special populations, such as HIV-HCV coinfected patients can be treated without need of fibrosis staging. Patients can be treated with a specific regimen during 12 or 24 weeks according to genotype, previous response, and presence of cirrhosis (Table 2). Due to the short period since implementation, the effectiveness of these new Brazilian guidelines was still not evaluated.

The access to DAAs is still incipient across Latin American countries. The process for introduction of DAAs in public health systems has just started in Mexico, Venezuela, Chile, and Argentina [22], Colombia has unofficial guideline from the local society of hepatology recommending the use of these drugs. Furthermore, Brazil is the first country where these innovative treatments could be recommended in officially published guidelines [16]. This situation is somewhat different from North America, where Canada and the United States of America already have interferon-free therapies in their national guidelines [100, 101].

Resistance to HCV treatment with Peg-interferon has been previously studied in Brazilian cohorts [102, 103]. The high specificity of DAAs against their viral targets might result in emergence of antiviral resistance and treatment failure in some patients. Resistance-associated mutants may arise prior to or during therapy and cross-resistance among DAAs is high, with resistance to one drug often conferring at least partial resistance to other drugs in the same class. Although methods for detecting resistance-associated variants (RAVs) have been described and commercial assays are available for certain variants (e.g., NS3 Q80K), there is no standard recommendation to evaluate patients for the presence of RAVs in clinical practice and there are few guidelines on their effective use [104]. Phase 3 studies and real-world experience have been showing that treatment using DAAs seems to be safe with very low rates of serious adverse effects [105].

The growing complexity of CHC treatment may reach a future point where a new specialty could emerge just to treat people living with HCV [106]. Due to this scenario, the effort to synthetize scientific knowledge into clear and evidence-based guidelines should be made [16, 100, 107].

New treatment regimens with DAAs are highly effective and safe, have short duration, and involve simple interferon-free oral coformulations, most of which without association of Ribavirin. However, these regimens are costly which might lead to a restricting societal benefit. Health policies might be implemented based on cost-effectiveness, stage of disease, and potential gain from treatment.

## 9. Conclusions

An estimated number of 1.45 million people are living with chronic hepatitis C in Brazil, representing a major public health issue. This liver disease presents a variable natural history ranging from minimal changes to advanced fibrosis and cirrhosis and its complications. Liver fibrosis might be accurately staged by noninvasive methods and new antiviral treatments might eradicate the virus in high rates. However, further research is needed to analyze the cost-effectiveness of noninvasive diagnostic strategies and prompt treatment with new antiviral drugs in the perspective of Brazilian universal health system.

## Figures and Tables

**Table 1:** Noninvasive methods for staging fibrosis in chronic hepatitis C.

Noninvasive method for estimation of liver fibrosis	Parameters
Biomarkers	
APRI	AST and platelet count
ELF	PIIINP, TIMP-1, and hyaluronic acid
FIB-4	Platelet count, ALT and AST adjusted for age
FibroIndex	platelet count, AST, and gamma-globulin
FibroMeter	Alpha-2-macroglobulin, hyaluronic acid, platelet count, AST, prothrombin time, and urea adjusted for age
FibroSpect II	Hyaluronic acid, TIMP-1, and alpha-2-macroglobulin
FibroTest	Apolipoprotein A1, haptoglobin, alpha-2-macroglobulin, GGT, and total bilirubin adjusted for age and gender
Forns index	Platelet count, GGT, and total cholesterol adjusted for age and gender
HepaScore	Alpha-2-macroglobulin, GGT, total bilirubin, and hyaluronic acid adjusted for age and gender
Imaging techniques	
AixPlorer	Real-time shear wave elastography
ARFI	Elastography
Fibroscan	Transient elastography
Magnetic Resonance Imaging	Elastography and water-diffusion abnormalities

ALT, alanine aminotransferase; AST, aspartate aminotransferase; ARFI, Acoustic Radiation Force Impulse; ELF, enhanced liver fibrosis; GGT, gamma-glutamyl transferase; PIIINP, serum amino-terminal propeptide of type III procollagen; TIMP-1, tissue inhibitors of matrix metalloproteinase.

**Table 2:** Guidelines for CHC treatment according to Brazilian Ministry of Health.

Conditions	Treatment	Duration
*Genotype 1*		
HCV monoinfected	Sofosbuvir + Simeprevir OR Sofosbuvir + Daclatasvir	12 weeks
HCV monoinfected with Child-Pugh B/C OR previously treated by Telaprevir/Boceprevir OR HIV-HCV coinfection	Sofosbuvir + Daclatasvir	24 weeks
*Genotype 2*		
HCV monoinfected	Sofosbuvir + Ribavirin	12 weeks
*Genotype 3*		
No contraindication for Peg-interferon	Sofosbuvir + Peg-interferon + Ribavirin	12 weeks
Contraindication for Peg-interferon	Sofosbuvir + Daclatasvir	12 weeks
*Genotype 4*		
No contraindication for Peg-interferon	Daclatasvir + Peg-interferon + Ribavirin	24 weeks
Contraindication for Peg-interferon	Sofosbuvir + Daclatasvir	12 weeks

Source: Brazilian Ministry of Health [16].

## References

[1] Alter M. J., Margolis H. S., Krawczynski K. (1992). The natural history of community-acquired hepatitis C in the United States. *The New England Journal of Medicine*.

[2] Pawlotsky J.-M. (2006). Virology of hepatitis B and C viruses and antiviral targets. *Journal of Hepatology*.

[3] Terrault N. A., Dodge J. L., Murphy E. L. (2013). Sexual transmission of hepatitis C virus among monogamous heterosexual couples: the HCV partners study. *Hepatology*.

[4] National Institutes of Health (2002). National institutes of health consensus development conference statement: management of hepatitis C: 2002—June 10–12, 2002. *Hepatology*.

[5] Reggiardo M. V., Fay F., Tanno M. (2012). Natural history of hepatitis c virus infection in a cohort of asymptomatic post-transfused subjects. *Annals of Hepatology*.

[6] European Association for Study of the Liver (2014). *EASL Recommendations on Treatment of Hepatitis C 2014*.

[7] Maity S., Nandi S., Biswas S., Sadhukhan S. K., Saha M. K. (2012). Performance and diagnostic usefulness of commercially available enzyme linked immunosorbent assay and rapid kits for detection of HIV, HBV and HCV in India. *Virology Journal*.

[8] Smith B. D., Drobeniuc J., Jewett A. (2011). Evaluation of three rapid screening assays for detection of antibodies to hepatitis C virus. *Journal of Infectious Diseases*.

[9] Moyer V. A. (2013). Screening for hepatitis C virus infection in adults: U.S. preventive services task force recommendation statement. *Annals of Internal Medicine*.

[10] Liang T. J., Ghany M. G. (2013). Current and future therapies for hepatitis C virus infection. *The New England Journal of Medicine*.

[11] Murphy D. G., Willems B., Deschênes M., Hilzenrat N., Mousseau R., Sabbah S. (2007). Use of sequence analysis of the NS5B region for routine genotyping of hepatitis C virus with reference to C/E1 and 5′ untranslated region sequences. *Journal of Clinical Microbiology*.

[12] Sy T., Jamal M. M. (2006). Epidemiology of hepatitis C virus (HCV) infection. *International Journal of Medical Sciences*.

[13] Abrantes J., Torres D. S., de Mello C. E. B. (2013). Patients with hepatitis C infection and normal liver function: an evaluation of cognitive function. *Postgraduate Medical Journal*.

[14] Vigani A. G., Pavan M. H., Tozzo R. (2008). Comparative study of patients with chronic hepatitis C virus infection due to genotypes 1 and 3 referred for treatment in southeast Brazil. *BMC Infectious Diseases*.

[15] Das B. R., Kundu B., Khandapkar R., Sahni S. (2002). Geographical distribution of hepatitis C virus genotypes in India. *Indian Journal of Pathology and Microbiology*.

[16] Ministério da Saúde. Secretaria de Vigilância em Saúde. Departamento de DST. Aids e Hepatites Virais (2015). *Protocolo Clínico e Diretrizes Terapêuticas para Hepatite C e Coinfecções*.

[17] Ansaldi F., Orsi A., Sticchi L., Bruzzone B., Icardi G. (2014). Hepatitis C virus in the new era: perspectives in epidemiology, prevention, diagnostics and predictors of response to therapy. *World Journal of Gastroenterology*.

[18] Lavanchy D. (2009). The global burden of hepatitis C. *Liver International*.

[19] Brasil. Ministério da Saúde (2012). *Boletim Epidemiológico—Hepatites Virais*.

[20] Ministério da Saúde (2015). *Hepatites Virais Casos Confirmados Notificados no Sistema de Informação de Agravos de Notificação por Ano de Diagnóstico/Sintomas*.

[21] Brasil. Ministério da Saúde (2015). *Boletim Epidemiológico—Hepatites Virais*.

[22] Botero R. C., Tagle M. (2015). New therapies for hepatitis C: Latin American perspectives. *Clinical Liver Disease*.

[23] Pereira L. M. M. B., Martelli C. M. T., Moreira R. C. (2013). Prevalence and risk factors of Hepatitis C virus infection in Brazil, 2005 through 2009: a Cross-Sectional Study. *BMC Infectious Diseases*.

[24] Fernandes F. F., Ferraz M. L., Andrade L. E. (2015). Enhanced liver fibrosis panel as a predictor of liver fibrosis in chronic hepatitis C patients. *Journal of Clinical Gastroenterology*.

[25] Perazzo H., Fernandes F. F., Gomes A., Terra C., Perez R. M., Figueiredo F. A. F. (2015). Interobserver variability in transient elastography analysis of patients with chronic hepatitis C. *Liver International*.

[26] Paranaguá-Vezozzo D. C., Ono S. K., Alvarado-Mora M. V. (2014). Epidemiology of HCC in Brazil: incidence and risk factors in a ten-year cohort. *Annals of Hepatology*.

[27] Akamatsu N., Sugawara Y. (2012). Liver transplantation and Hepatitis C. *International Journal of Hepatology*.

[28] Klaus F., Keitel da Silva C., Meinerz G. (2014). Acute kidney injury after liver transplantation: incidence and mortality. *Transplantation Proceedings*.

[29] Salvalaggio P. R., Caicedo J. C., de Albuquerque L. C. (2014). Liver transplantation in Latin America: the state-of-the-art and future trends. *Transplantation*.

[30] Barrera J. M., Bruguera M., Guadalupe Ercilla M. (1995). Persistent hepatitis C viremia after acute self-limiting posttransfusion hepatitis C. *Hepatology*.

[31] Poynard T., Ratziu V., Benmanov Y., Di Martino V., Bedossa P., Opolon P. (2000). Fibrosis in patients with chronic hepatitis C: detection and significance. *Seminars in Liver Disease*.

[32] Villano S. A., Vlahov D., Nelson K. E., Cohn S., Thomas D. L. (1999). Persistence of viremia and the importance of long-term follow-up after acute hepatitis C infection. *Hepatology*.

[33] Poynard T., Bedossa P., Opolon P. (1997). Natural history of liver fibrosis progression in patients with chronic hepatitis C. *The Lancet*.

[34] Alter H. J., Seeff L. B. (2000). Recovery, persistence, and sequelae in hepatitis C virus infection: a perspective on long-term outcome. *Seminars in Liver Disease*.

[35] Cohen P. (2000). Extrahepatic manifestations of the hepatitis C virus. *Presse Medicale*.

[36] Prati D., Zanella A., De Mattei C. (1999). Chronic hepatitis C virus infection and primary cutaneous B-cell lymphoma. *British Journal of Haematology*.

[37] Planas R., Ballesté B., Álvarez M. A. (2004). Natural history of decompensated hepatitis C virus-related cirrhosis. A study of 200 patients. *Journal of Hepatology*.

[38] Chen S. L., Morgan T. R. (2006). The natural history of hepatitis C virus (HCV) infection. *International Journal of Medical Sciences*.

[39] Bedossa P. (1994). Intraobserver and interobserver variations in liver biopsy interpretation in patients with chronic hepatitis C. *Hepatology*.

[40] Ishak K., Baptista A., Bianchi L. (1995). Histological grading and staging of chronic hepatitis. *Journal of Hepatology*.

[41] McGill D. B., Rakela J., Zinsmeister A. R., Ott B. J. (1990). A 21-year experience with major hemorrhage after percutaneous liver biopsy. *Gastroenterology*.

[42] Bergesch D'Incao R., da Silva M. C. A., de Almeida P. R. L., Plasse Renon V., Tovo C. V. (2013). Percutaneous liver biopsy—2 decades of experience in a public hospital in the South of Brazil. *Annals of Hepatology*.

[43] Bravo A. A., Sheth S. G., Chopra S. (2001). Liver biopsy. *The New England Journal of Medicine*.

[44] Bedossa P., Dargère D., Paradis V. (2003). Sampling variability of liver fibrosis in chronic hepatitis C. *Hepatology*.

[45] Rousselet M.-C., Michalak S., Dupré F. (2005). Sources of variability in histological scoring of chronic viral hepatitis. *Hepatology*.

[46] Poynard T., Morra R., Ingiliz P. (2008). Assessment of liver fibrosis: noninvasive means. *Saudi Journal of Gastroenterology*.

[47] Sharma S., Khalili K., Nguyen G. C. (2014). Non-invasive diagnosis of advanced fibrosis and cirrhosis. *World Journal of Gastroenterology*.

[48] Bota S., Herkner H., Sporea I. (2013). Meta-analysis: ARFI elastography versus transient elastography for the evaluation of liver fibrosis. *Liver International*.

[49] Wai C.-T., Greenson J. K., Fontana R. J. (2003). A simple noninvasive index can predict both significant fibrosis and cirrhosis in patients with chronic hepatitis C. *Hepatology*.

[50] Perazzo H., Pais R., Munteanu M. (2014). Variability in definitions of transaminase upper limit of the normal impacts the APRI performance as a biomarker of fibrosis in patients with chronic hepatitis C: ‘APRI c'est fini?’. *Clinics and Research in Hepatology and Gastroenterology*.

[51] Chou R., Wasson N. (2013). Blood tests to diagnose fibrosis or cirrhosis in patients with chronic hepatitis C virus infection: a systematic review. *Annals of Internal Medicine*.

[52] Nunes D., Fleming C., Offner G. (2010). Noninvasive markers of liver fibrosis are highly predictive of liver-related death in a cohort of HCV-infected individuals with and without HIV infection. *American Journal of Gastroenterology*.

[53] Vergniol J., Foucher J., Terrebonne E. (2011). Noninvasive tests for fibrosis and liver stiffness predict 5-year outcomes of patients with chronic hepatitis C. *Gastroenterology*.

[54] Poynard T., Ngo Y., Perazzo H. (2011). Prognostic value of liver fibrosis biomarkers: a meta-analysis. *Gastroenterology & Hepatology*.

[55] Parise E. R., Oliveira A. C., Figueiredo-Mendes C. (2006). Noninvasive serum markers in the diagnosis of structural liver damage in chronic hepatitis C virus infection. *Liver International*.

[56] da Silva R. G., Fakhouri R., do Nascimento T. V. B., Santos I. M., Barbosa L. M. M. (2008). Aspartate aminotransferase-to-platelet ratio index for fibrosis and cirrhosis prediction in chronic hepatitis C patients. *Brazilian Journal of Infectious Diseases*.

[57] Amorim T. G. F., Staub G. J., Lazzarotto C. (2012). Validation and comparison of simple noninvasive models for the prediction of liver fibrosis in chronic hepatitis C. *Annals of Hepatology*.

[58] Silva Junior R. G., Schmillevitch J., Nascimento M. D. F. A. (2014). Acoustic radiation force impulse elastography and serum fibrosis markers in chronic hepatitis C. *Scandinavian Journal of Gastroenterology*.

[59] de Lédinghen V., Vergniol J. (2008). Transient elastography (FibroScan). *Gastroenterologie Clinique et Biologique*.

[60] Perazzo H., Fernandes F. F., Soares J. C. (2015). Learning curve and intra/interobserver agreement of transient elastography in chronic hepatitis C patients with or without HIV co-infection. *Clinics and Research in Hepatology and Gastroenterology*.

[61] Castéra L., Vergniol J., Foucher J. (2005). Prospective comparison of transient elastography, Fibrotest, APRI, and liver biopsy for the assessment of fibrosis in chronic hepatitis C. *Gastroenterology*.

[62] Ziol M., Handra-Luca A., Kettaneh A. (2005). Noninvasive assessment of liver fibrosis by measurement of stiffness in patients with chronic hepatitis C. *Hepatology*.

[63] Poynard T., Vergniol J., Ngo Y. (2014). Staging chronic hepatitis C in seven categories using fibrosis biomarker (FibroTest) and transient elastography (FibroScan). *Journal of Hepatology*.

[64] Alves G. A., Baldessar M. Z., Pereira G. W., Kuehlkamp V. M., Hilzendeger C., da Silva J. (2012). Quality of life of patients with hepatitis C. *Revista da Sociedade Brasileira de Medicina Tropical*.

[65] El Khoury A. C., Vietri J., Prajapati G. (2014). Health-related quality of life in patients with hepatitis C virus infection in Brazil. *Pan American Journal of Public Health*.

[66] Strauss E., Porto-Ferreira F. A., de Almeida-Neto C., Teixeira M. C. D. (2014). Altered quality of life in the early stages of chronic hepatitis C is due to the virus itself. *Clinics and Research in Hepatology and Gastroenterology*.

[67] Teixeira M. C. D., Ribeiro M. D. F. G. S., Gayotto L. C. D. C., Chamone D. D. A. F., Strauss E. (2006). Worse quality of life in volunteer blood donors with hepatitis C. *Transfusion (Paris)*.

[68] Fábregas B. C., de Ávila R. E., Faria M. N., Moura A. S., Carmo R. A., Teixeira A. L. (2013). Health related quality of life among patients with chronic hepatitis C: a cross-sectional study of sociodemographic, psychopathological and psychiatric determinants. *Brazilian Journal of Infectious Diseases*.

[69] Fábregas B. C., Moura A. S., de Ávila R. E., Faria M. N., Carmo R. A., Teixeira A. L. (2014). Sexual dysfunction and dissatisfaction in chronic hepatitis C patients. *Revista da Sociedade Brasileira de Medicina Tropical*.

[70] Morais-de-Jesus M., Daltro-Oliveira R., Pettersen K. M. (2014). Hepatitis C virus infection as a traumatic experience. *PLoS ONE*.

[71] Fonseca M. C. M., de Araújo G. T. B., Araújo D. V. (2009). Cost effectiveness of peginterferon alfa-2B combined with ribavirin for the treatment of chronic hepatitis C in Brazil. *Brazilian Journal of Infectious Diseases*.

[72] Barros F. M. R., Cheinquer H., Tsuchiya C. T., Santos E. A. V. (2013). Cost-effectiveness analysis of treatment with peginterferon-alfa-2a versus peginterferon-alfa-2b for patients with chronic hepatitis C under the public payer perspective in Brazil. *Cost Effectiveness and Resource Allocation*.

[73] Massad E., Coutinho F. A. B., Chaib E., Burattini M. N. (2009). Cost-effectiveness analysis of a hypothetical hepatitis C vaccine compared to antiviral therapy. *Epidemiology and Infection*.

[74] da S. Rodrigues M. P., de M. Vianna C. M., Mosegui G. B. G., Costa e Silva F. V., de F. Peregrino A. A., Jardim F. N. (2013). Cost-effectiveness of hepatitis C treatment in slow virologic responders coinfected with HIV. *Cadernos de Saúde Pública*.

[75] Bichoupan K., Martel-Laferriere V., Sachs D. (2014). Costs of telaprevir-based triple therapy for hepatitis C: $189,000 per sustained virological response. *Hepatology*.

[76] van der Meer A. J., Veldt B. J., Feld J. J. (2012). Association between sustained virological response and all-cause mortality among patients with chronic hepatitis C and advanced hepatic fibrosis. *The Journal of the American Medical Association*.

[77] Imran M., Manzoor S., Ashraf J. (2013). Role of viral and host factors in interferon based therapy of hepatitis C virus infection. *Virology Journal*.

[78] Chevaliez S. (2011). Antiviral activity of the new DAAs for the treatment of hepatitis C virus infection: virology and resistance. *Clinics and Research in Hepatology and Gastroenterology*.

[79] Artico S., Amaral K. M., Gonçalves C. B. T., Picon P. D. (2012). The effectiveness of retreatment with peginterferon alfa and ribavirin in patients with chronic viral hepatitis C genotype 2 and 3: a prospective cohort study in Brazil. *BMC Infectious Diseases*.

[80] Vigani A. G., Gonçales E. S., Pavan M. H. P. (2012). Therapeutic effectiveness of biosimilar standard interferon versus pegylated interferon for chronic hepatitis C genotypes 2 or 3. *Brazilian Journal of Infectious Diseases*.

[81] Webster D. P., Klenerman P., Dusheiko G. M. (2015). Hepatitis C. *The Lancet*.

[82] Poordad F., McCone J., Bacon B. R. (2011). Boceprevir for untreated chronic HCV genotype 1 infection. *The New England Journal of Medicine*.

[83] Jacobson I. M., McHutchison J. G., Dusheiko G. (2011). Telaprevir for previously untreated chronic hepatitis C virus infection. *The New England Journal of Medicine*.

[84] Hézode C., Fontaine H., Dorival C. (2014). Effectiveness of telaprevir or boceprevir in treatment-experienced patients with HCV genotype 1 infection and cirrhosis. *Gastroenterology*.

[85] Kiser J. J., Burton J. R., Everson G. T. (2013). Drug-drug interactions during antiviral therapy for chronic hepatitis C. *Nature Reviews Gastroenterology and Hepatology*.

[86] Pawlotsky J.-M. (2011). Treatment failure and resistance with direct-acting antiviral drugs against hepatitis C virus. *Hepatology*.

[87] Manns M., Marcellin P., Poordad F. (2014). Simeprevir with pegylated interferon alfa 2a or 2b plus ribavirin in treatment-naive patients with chronic hepatitis C virus genotype 1 infection (QUEST-2): a randomised, double-blind, placebo-controlled phase 3 trial. *The Lancet*.

[88] Kowdley K. V., Lawitz E., Crespo I. (2013). Sofosbuvir with pegylated interferon alfa-2a and ribavirin for treatment-naive patients with hepatitis C genotype-1 infection (ATOMIC): an open-label, randomised, multicentre phase 2 trial. *The Lancet*.

[89] Afdhal N., Reddy K. R., Nelson D. R. (2014). Ledipasvir and sofosbuvir for previously treated HCV genotype 1 infection. *The New England Journal of Medicine*.

[90] Kowdley K. V., Gordon S. C., Reddy K. R. (2014). Ledipasvir and sofosbuvir for 8 or 12 weeks for chronic HCV without cirrhosis. *The New England Journal of Medicine*.

[91] Zeuzem S., Jacobson I. M., Baykal T. (2014). Retreatment of HCV with ABT-450/r—ombitasvir and dasabuvir with ribavirin. *The New England Journal of Medicine*.

[92] Poordad F., Hezode C., Trinh R. (2014). ABT-450/r-ombitasvir and dasabuvir with ribavirin for hepatitis C with cirrhosis. *The New England Journal of Medicine*.

[93] Lawitz E., Sulkowski M. S., Ghalib R. (2014). Simeprevir plus sofosbuvir, with or without ribavirin, to treat chronic infection with hepatitis C virus genotype 1 in non-responders to pegylated interferon and ribavirin and treatment-naive patients: the COSMOS randomised study. *The Lancet*.

[94] Jacobson I. M., Gordon S. C., Kowdley K. V. (2013). Sofosbuvir for hepatitis C genotype 2 or 3 in patients without treatment options. *The New England Journal of Medicine*.

[95] Zeuzem S., Dusheiko G. M., Salupere R. (2014). Sofosbuvir and Ribavirin in HCV genotypes 2 and 3. *The New England Journal of Medicine*.

[96] Nelson D. R., Cooper J. N., Lalezari J. P. (2015). All-oral 12-week treatment with daclatasvir plus sofosbuvir in patients with hepatitis C virus genotype 3 infection: ALLY-3 phase III study. *Hepatology*.

[97] Gane E. J., Hyland R. H., An D. (2014). Sofosbuvir/ledipasvir fixed dose combination is safe and effective in difficult-to-treat populations including genotype-3 patients, decompensated genotype-1 patients, and genotype-1 patients with prior sofosbuvir treatment experience. *Journal of Hepatology*.

[98] Ruane P. J., Ain D., Riad J. Sofosbuvir plus ribavirin in the treatment of chronic HCV genotype 4 infection in patients of Egyptian ancestry.

[99] Hezode C., Marcellin P., Pol S. (2014). Results from the phase 2 PEARL-I study: interferon-free regimens of ABT-450/R + ABT-267 with or without ribavirin in patients with HCV genotype 4 infection. *Journal of Hepatology*.

[100] Myers R. P., Shah H., Burak K. W., Cooper C., Feld J. J. (2015). An update on the management of chronic hepatitis C: 2015 Consensus guidelines from the Canadian Association for the Study of the Liver. *Canadian Journal of Gastroenterology and Hepatology*.

[101] AASLD/IDSA HCV Guidance Panel (2015). Hepatitis C guidance: AASLD-IDSA recommendations for testing, managing, and treating adults infected with hepatitis C virus. *Hepatology*.

[102] Hoffmann L., Ramos J. A., de Souza E. V. (2013). Dynamics of resistance mutations to NS3 protease inhibitors in a cohort of Brazilian patients chronically infected with hepatitis C virus (genotype 1) treated with pegylated interferon and ribavirin: a prospective longitudinal study. *Virology Journal*.

[103] Zeminian L. B., Padovani J. L., Corvino S. M., Silva G. F., Pardini M. I. D. M. C., Grotto R. M. T. (2013). Variability and resistance mutations in the hepatitis C virus NS3 protease in patients not treated with protease inhibitors. *Memórias do Instituto Oswaldo Cruz*.

[104] Sarrazin C. (2015). The importance of resistance to direct antiviral drugs in HCV infection in clinical practice. *Journal of Hepatology*.

[105] Bansal S., Singal A. K., McGuire B. M., Anand B. S. (2015). Impact of all oral anti-hepatitis C virus therapy: a meta-analysis. *World Journal of Hepatology*.

[106] Soriano V., Vispo E., Poveda E. (2011). Directly acting antivirals against hepatitis C virus. *Journal of Antimicrobial Chemotherapy*.

[107] Booth J. C., O'Grady J., Neuberger J., Thr Royal College of Physicians of London and the British Society of Gastroenterology (2001). Clinical guidelines on the management of hepatitis C. *Gut*.

